# Supplementary material for: Disclosing Potential Key Genes, Therapeutic Targets and Agents for Non-Small Cell Lung Cancer: Evidence from Integrative Bioinformatics Analysis
Source: Vaccines (Basel). 2022 May 12;10(5):771. doi: 10.3390/vaccines10050771 (PMC9143695; doi:10.3390/vaccines10050771)
Supplement: Supplementary file 1 [file vaccines-10-00771-s001.zip › vaccines-1647540-supplementary.pdf]

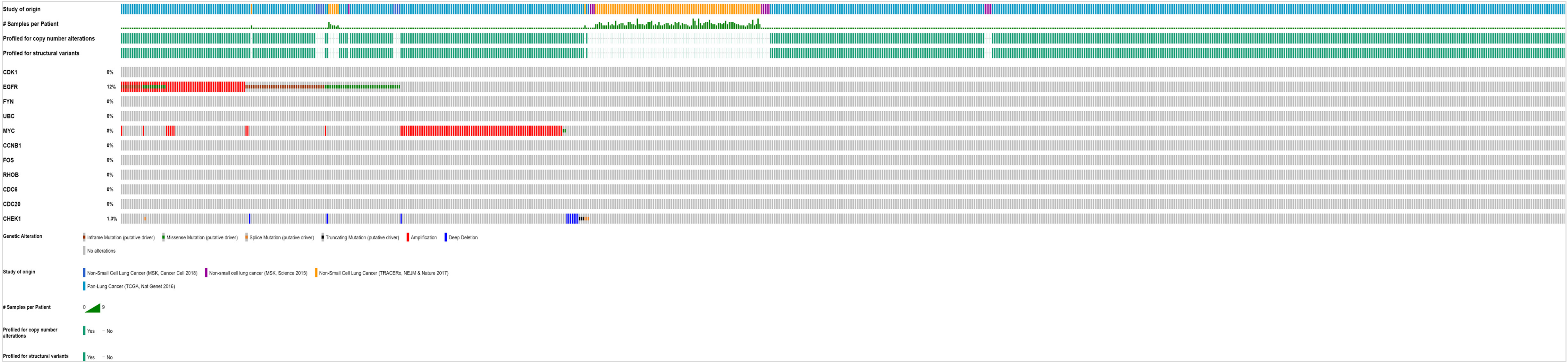
Study of origin
Samples per Patient
Profiled for copy number alterations
Profiled for structural variants
CDK1 0%
EGFR 12%
FYN 0%
UBC 0%
MYC 8%
CCNB1 0%
FOS 0%
RHOB 0%
CDC6 0%
CDC20 0%
CHEK1 1.3%
Genetic Alteration
Inframe Mutation (putative driver)
Missense Mutation (putative driver)
Splice Mutation (putative driver)
Truncating Mutation (putative driver)
Amplification
Deep Deletion
No alterations
Study of origin
Non-Small Cell Lung Cancer (MSK, Cancer Cell 2018)
Non-small cell lung cancer (MSK, Science 2015)
Non-Small Cell Lung Cancer (TRACERx, NEJM & Nature 2017)
Pan-Lung Cancer (TCGA, Nat Genet 2016)
Samples per Patient
0
9
Profiled for copy number alterations
Yes
No
Profiled for structural variants
Yes
No

(A)

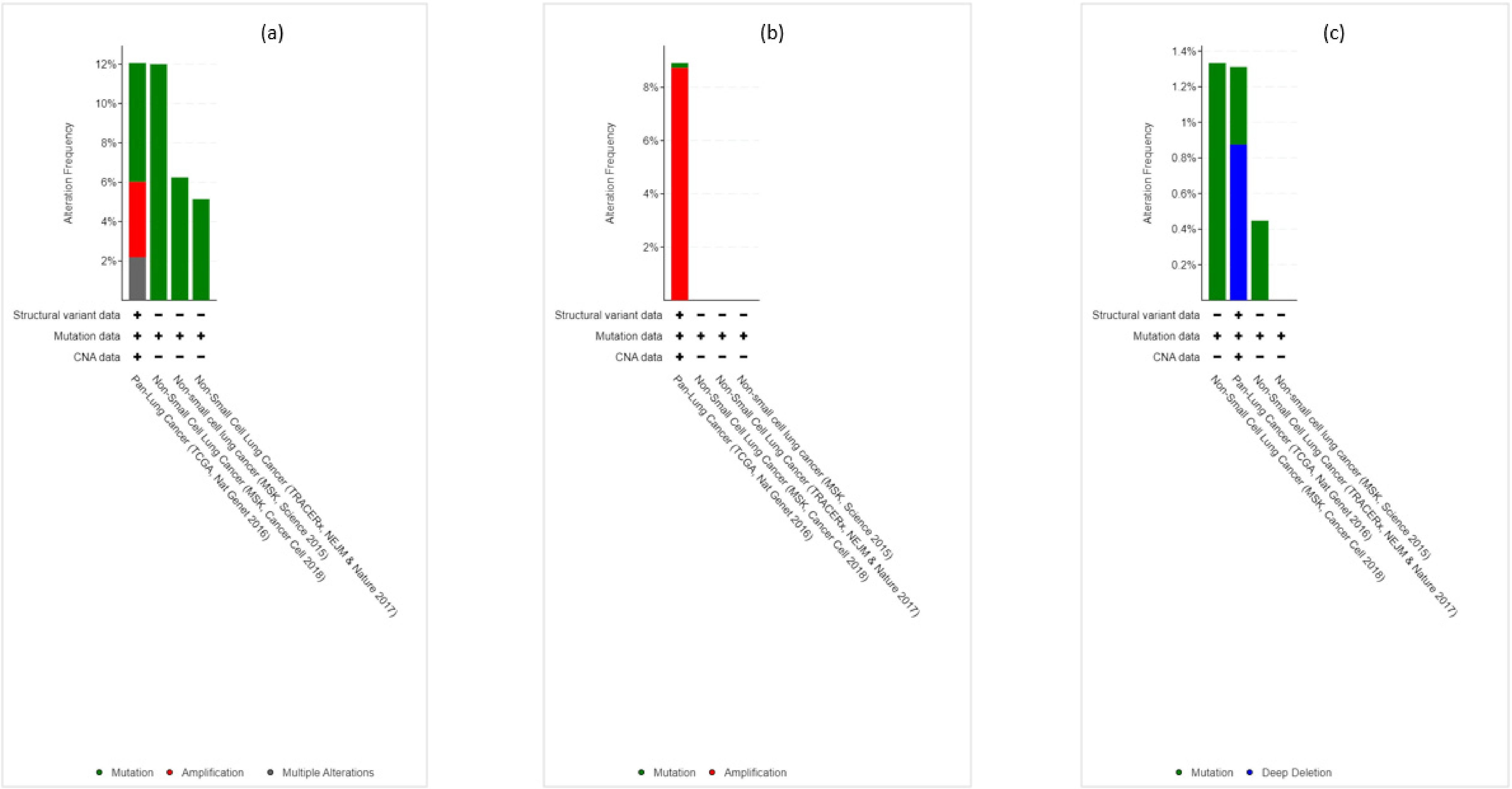

(B)

Figure S1. (A): The genomic alteration of the proposed 11 hub-DEGs among the NSCLC. The genomic mutations of the proposed genes are represented here where the infrared red color denotes the "inframe mutation", infrared green color denotes "missense mutation" of known significance, infrared yellow color denotes "splice mutation", infrared grey color denotes "Truncating mutation" of unknown significance, red color denotes "amplification" and blue color denotes "deep deletion" of unknown significance. (B) (a) EGFR (b) MYC and (c) CHEK1 alteration frequency over the four lung cancer studies.
